# A covalent homodimer probing early oligomers along amyloid aggregation

**DOI:** 10.1038/srep14651

**Published:** 2015-09-30

**Authors:** Levon Halabelian, Annalisa Relini, Alberto Barbiroli, Amanda Penco, Martino Bolognesi, Stefano Ricagno

**Affiliations:** 1 Dipartimento di Bioscienze, Università degli Studi di Milano, Via Celoria 26, 20133 Milan, Italy; 2 Dipartimento di Fisica, Università di Genova, via Dodecaneso 33, 16146 Genova, Italy; 3 Dipartimento di Scienze per gli Alimenti, la Nutrizione e l’Ambiente, Università degli Studi di Milano, via Celoria 2, 20133 Milan, Italy; 4 CIMAINA e Istituto CNR di Biofisica, Milano, Italy

## Abstract

Early oligomers are crucial in amyloid aggregation; however, due to their transient nature they are among the least structurally characterized species. We focused on the amyloidogenic protein beta2-microglobulin (β2m) whose early oligomers are still a matter of debate. An intermolecular interaction between D strands of facing β2m molecules was repeatedly observed, suggesting that such interface may be relevant for β2m dimerization. In this study, by mutating Ser33 to Cys, and assembling the disulphide-stabilized β2m homodimer (DimC33), such DD strand interface was locked. Although the isolated DimC33 display a stability similar to wt β2m under native conditions, it shows enhanced amyloid aggregation propensity. Three distinct crystal structures of DimC33 suggest that dimerization through the DD interface is instrumental for enhancing DimC33 aggregation propensity. Furthermore, the crystal structure of DimC33 in complex with the amyloid-specific dye Thioflavin-T pinpoints a second interface, which likely participates in the first steps of β2m aggregation. The present data provide new insight into β2m early steps of amyloid aggregation.

Amyloidogenesis is a complex and non-homogeneous process whereby, during the nucleation phase, monomeric protein molecules start to associate, firstly yielding oligomeric species that eventually lead to mature amyloid fibrils^1^. The mechanism of amyloid toxicity is still debated, although several experimental evidences suggest that pre-fibrillar oligomeric species might play a crucial role in determining cytotoxicity and tissue sufferance^2^. Shedding light on the molecular bases of oligomer assembly is therefore relevant to understand the specific properties of the cytotoxic species. Nevertheless, since the oligomers are transient species in equilibrium with higher/lower molecular weight aggregates, only in few cases they have been successfully isolated^3^. Even when isolated *in vitro*, typically the oligomers are too heterogeneous and unstable for high-resolution structural investigations. Accordingly, an experimental description of oligomers at the molecular level is generally lacking, most evidences on oligomer structure being obtained though spectroscopic techniques or microscopy investigations^4^. To date computational approaches, such as molecular dynamics simulations, are producing increasingly reliable models of protein oligomerisation^5^,^6^.

In order to elucidate the structural bases of oligomer formation we focused on the amyloidogenic protein beta-2 microglobulin (β2m), whose native fold is structurally well characterized, and amyloid formation *in vitro* has been thoroughly described^7^. β2m is a 99-residue globular protein with a typical immunoglobulin-like fold, composed of seven β-strands arranged in two β-sheets, named ABDE and CFG, respectively, according to standard nomenclature of the composing β-strands (Fig. 1a); the two sheets are internally linked by a disulphide bond^8^. β2m is an aggregation-prone protein responsible for two types of amyloid-related diseases: the wild type (wt) protein is responsible for Dialysis-Related Amyloidosis (DRA)^9^, while a severe hereditary systemic amyloidosis is linked to the pathological β2m D76N mutant^10^. Physiologically, β2m is degraded in the kidneys; DRA patients typically suffer from kidney dysfunction that results in β2m accumulation in the serum following dialysis. Over the years, β2m aggregates in the skeletal joints, bones and muscles, resulting in bone fragility and movement impairment^11^.

Wt β2m aggregation propensity has been extensively studied in the last decades, although one important limitation for the study of β2m aggregation is that the wt protein *in vitro* is stable for months under native conditions. Accordingly, all wt β2m aggregation protocols are based on conditions under which the protein is completely or partially unfolded (low pH, denaturants such 2,2,2-trifluoroethanol (TFE), or sodium dodecylsulphate) and the addition of amyloid-fibril seeds to the reaction mixture is necessary^12^. Thus, such *in vitro* protocols may not describe the natural β2m oligomerisation occurring *in vivo*, where the protein is unlikely to be largely unfolded before amyloid deposition takes place. Moreover, the addition of seeds allows to study fibril growth, but it fully abolishes the key early process of oligomer formation.

Several independent reports suggest that the first oligomeric species formed during β2m aggregation is an elongated dimer: extended oligomers have been observed by mass-spectrometry, and an elongated dimer was proposed to be the starting species in β2m oligomerisation^13^,^14^,^15^,^16^. Among several studies aimed at uncovering the structure of β2m oligomers^17^,^18^, Miranker and coworkers reported the crystal structure of a hexameric form of the H13F β2m mutant^17^, formed under aggregating conditions; such hexameric assembly is however not amyloidogenic^17^.

We recently tested a different approach to the study of β2m oligomer formation. Single Cys mutations were inserted in different β2m surface regions, and disulphide-linked covalent homodimers were prepared^19^. The rationale was that a covalent bond between two β2m molecules acts as a constraint for the architecture of the dimer, which can be aggregation permissive or non-permissive. The crystal structures of two of such dimers showed a common antiparallel interface between covalent dimers (named the DD strand interface): these dimers also proved to be amyloidogenic and assembled as dimer of dimers in solution. Notably the S-S bond location in a third engineered homodimer hampers the formation of the DD strand interface resulting in a non-amyloidogenic, purely dimeric species in solution. Based on such observations, the DD strand interface was proposed as a key association interface involved in the early stages of β2m association under native and non-native conditions^19^.

The DD strand interface involves the apical region of two β2m molecules. The D-strands belonging to the facing molecules are antiparallel and the two β2m molecules involved display the same conformation and are well superposable. Within each β2m monomer the DD strand interface involves the D-strand, the DE and BC loops, namely regions that have been reported as major players in β2m amyloid aggregation^19^ (Fig. 1). Such intermolecular association interface had also been noted in the hexameric structure of the H13F β2m mutant^17^.

Following our previous approach, in the present study we engineered a β2m homodimer where the DD strand interface is specifically locked by a disulphide bond (linking the engineered C33 residues of two β2m molecules – DimC33). We speculated that analysis of such a covalently stabilized dimer should allow us to assess more directly the role played by association through the DD strand interface in β2m oligomerisation and amyloid formation. DimC33 has been characterized in solution and under aggregation conditions; moreover, we determined three different crystal structures of the covalent dimer, one of which hosts the DimC33 complex with Thioflavin (ThT), the hallmark fluorescent dye for amyloid aggregates. The data here reported show that a covalently stabilized DD strand interface facilitates β2m aggregation under denaturating and non-denaturating conditions,, suggesting that DimC33 may be used as a model system to study β2m early oligomerisation steps *in vitro*. Our results recapitulate previous data indicating the DD strand interface as the first and favourite intermolecular contact region between β2m molecules under native or aggregating conditions. Furthermore, analysis of the crystal structure of DimC33 in complex with ThT strongly points to a second β2m association interface that may be involved in amyloid aggregation.

## Results

### Recombinant S33C variant β2m homodimers

In order to engineer a β2m covalent dimer displaying a locked DD strand interface, a detailed analysis of the crystal structures displaying such intermolecular association interface (pdb codes: 3TM6, 3TLR, 3CIQ) was performed, and the mutation of Ser33 to Cys was chosen based on the following rationale. Firstly, two Ser33 residues belonging to two facing β2m molecules (in the examined crystal structures) fall at a suitable distance to be mutated to Cys and yield a disulphide bridge without disrupting or altering the DD interface. Secondly, among the residues, which could satisfy the above conditions, a Ser residue was chosen because it is essentially isosteric with Cys.

The S33C β2m variant was expressed and purified under denaturating conditions and then refolded, according to our standard protocols^20^. In our previous study on covalent β2m homodimers, in order to promote the formation of covalent disulphide linked homodimers, an oxidation reaction proved necessary. To this purpose, after refolding, the monomeric β2m variants were incubated at high protein concentrations in the presence of H_2_O_2_^19^, indicating that random encounters between molecules are not sufficient for the formation of disulphide linked dimers. Conversely, in the case of the S33C β2m variant, a covalent dimeric species was abundantly present at the end of the purification, in the absence of further oxidation reactions. The spontaneous formation of disulphide bonds greatly depends on proper juxtaposition of the two Cys residues involved^21^. Therefore, the unprompted dimerization of S33C implies that in solution, under native conditions, the DD association interface spontaneously brings together two monomeric β2m molecules, and only once the DD interface is properly assembled, the two facing Cys33 establish the disulphide bond, which results in the formation of the covalent dimeric species (DimC33).

The stability of DimC33 in solution was assessed by circular dichroism. Thermal unfolding monitored by Far-UV indicates that, as for wt β2m, DimC33 unfolds according to a simple sigmoidal curve and displays a Tm value close to that of the wt protein (Tm_DimC33_ 60.2 ± 0.3 °C; Tm_wt_ 62.4 ± 0.3 °C) (Fig. 2a). Then, Far-UV spectra of DimC33 and of wt β2m in phosphate buffer, in 10% and 20% TFE were collected, showing that native spectra are well superposable and that both proteins display a native secondary structure content in 10% TFE; conversely the β2m fold is perturbed in 20% TFE (Fig. 2b). Thus, the spectroscopic data suggest that the engineered mutation and the achievement of a covalent dimeric state do not alter β2m thermodynamic stability or its overall fold in DimC33.

### Aggregation kinetics of DimC33 and wt ß2m *in vitro*

We studied DimC33 aggregation propensity *in vitro* at physiological pH conditions (pH 7.4) in the absence of any pre-fibrillar seeds. In 20% (v/v) TFE, DimC33 at a final concentration of 1 mg mL^−1^ aggregates promptly without a lag phase, reaching equilibrium within the first 4 hours, and yielding a high ThT binding signal (Fig. 3a); under the same conditions wt β2m remains soluble and does not aggregate. Interestingly, in 10% (v/v) TFE—where DimC33 and wt β2m display CD spectra indicative of native-like conformations—a 5 mg mL^−1^ solution of DimC33 also aggregated. The AFM analysis of the samples after 1 week aggregation showed that DimC33 formed fibrils both in 20% TFE (Fig. 3b, left) and in 10% TFE (Fig. 3b, right). The fibril heights measured in the two conditions were similar and were in the range between 2.0 and 5.5 nm.

Doxycycline is a known inhibitor of wt β_2_m aggregation^22^. In order to assess doxycycline inhibitory effect on DimC33 aggregation, a DimC33 solution was tested for aggregation in the presence of 100 μM and 400 μM Doxycycline, in 20% TFE. A 100 μM final concentration of Doxycycline was sufficient to block DimC33 aggregation by approximately 85%, compared with a DimC33_20% TFE control solution, whereas 400 μM Doxycycline completely inhibited DimC33 aggregation (Fig. 3c). No fibrillar aggregates were detected by AFM in DimC33 samples incubated for 1 week in the presence of doxycycline (not shown). Such results are in keeping with previous data on wt protein reporting an IC50 of 47 μM for doxycycline as aggregation inhibitor^22^.

### X-ray crystal structures of DimC33

In order to assess fine details of the molecular structure of DimC33, two crystal structures of DimC33 were determined to 1.9 Å and 1.4 Å resolution, hereafter named DimC33_low and DimC33_high, respectively; additionally a crystal structure of DimC33 in complex with ThT was determined to 2.8 Å resolution, and named DimC33_ThT. Data collection and refinement statistics for the crystal structures are shown in Table 1.

**Figure 1:**
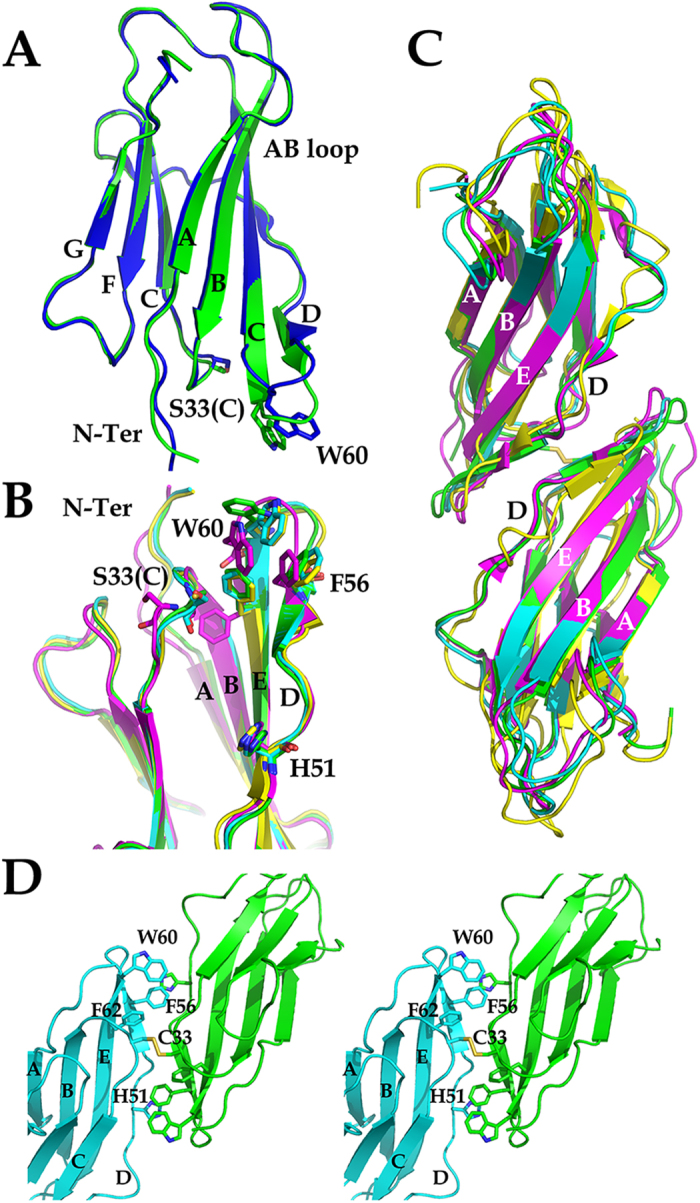
Crystal structure of DimC33 and DD strand interface. (**A**) Ribbon model of one of the two chains in DimC33 (blue) superposed on the structure of wt β2m (green). β-strands are labeled according to the standard β2m nomenclature, Ser33/Cys33 are shown in sticks. (**B**) A zoomed view into the DD interface of four superposed crystal structures of DimC33_ThT (green, pdb code: 4RA3), hexameric structure of H13F β2m (magenta, pdb code: 3CIQ), DimC50 (cyan, pdb code: 3TM6) and DimC20 (yellow, pdb code: 3TLR), showing the main residues involved in the DD interface as sticks model. (**C**) Superposition of three β2m non-covalently assembled dimers built through the DD strand interface with DimC33: DimC33_ThT in green, hexameric structure of H13F β2m in magenta, DimC50 structure in cyan, and DimC20 structure in yellow. (**D**) Stereo view of the DD strand interface built by the facing β2m molecules as observed in the structure of DimC33_high. The main residues involved in the interface are shown as sticks.

The three crystal structures display three different space groups and crystal packings. The electron density is of excellent quality and all β2m molecules are completely traceable in all three structures. Overall, no major structural effects on the β2m fold are induced by the S33C mutation, either locally or globally, in all DimC33 structures, (Fig. 1b,c). The monomeric components of DimC33 most closely match the conformation of wt β2m as observed in its physiologic class I major histocompatibility complex (Table 2). The only noticeable difference occurs in the DE loop (residues 57–60) that displays a slightly modified conformation so that Trp60 participates in stacking interactions with Phe56, of the same chain, and with His51 and Tyr66 of the second chain within DimC33; the rotation of Tyr63 side chain of about 90 degrees is also observed (see Fig. 1b).

The covalent DD strand interface is structurally well conserved in all three DimC33 structures, where it maintains the same contact surface of 560 Å^2^. The overall dimer organization is conserved (r.m.s.d. 0.57 Å/195 Cα, 1.17 Å/195 Cα, DimC33_high *versus* DimC33_ThT and *versus* DimC33_low, respectively); the main regions involved in the association interface are the BC loop (residues 31–37), the D strand (residues 51–57), the DE loop (residues 57–6), Phe62, the E strand (residues 64 and 66), as observed in previous structures (Fig. 1b–d). The superposition of DimC33 with previously reported β2m dimers, non-covalently associated through the DD strand interface, shows a very similar overall monomer-monomer orientation (Fig. 1c and Table 2) and conserved conformations for the residues involved (Fig. 1b–d and Table 2). All such data indicate that the S33C mutation minimally affects the β2m fold, and that the C33 – 33C disulphide covalently locks the DD strand interface with negligible structural effects Fig. 1b–d.

### The DimC33 ThT complex and the ABDE sheet

DimC33 was also crystallized in the presence of 5 mM ThT. Intriguingly, the crystallographic analysis showed that five ThT molecules are hosted in the crystal asymmetric unit (AU), which contains one DimC33 moiety and two halves of a second one (*i.e.* two additional and independent β2m chains). As a result of crystal packing, β2m molecules belonging to independent DimC33 units interact via their ABDE β-sheets (Fig. 4a); however, two distinct ABDE interfaces can be distinguished. In one of these, four ThT molecules are wedged between the ABDE sheets of two facing β2m molecules (4 ThT site); in the other, only one ThT molecule is sandwiched between two facing ABDE sheets (1 ThT site) (Fig. 4a). In either case, binding of the ThT molecules does not induce any conformational adjustments in the β2m fold, or in the DimC33 assembly. At the 1 ThT site, the ThT molecule is sandwiched between Tyr10 residues from two facing β2m molecules; moreover, Tyr26 and Pro14 from both molecules establish van der Waals interactions with ThT (Fig. 4b). At the 4 ThT site, the four ThT molecules are stacked on each other, and in stacking contacts with Tyr10 and Tyr63 of the β2m molecules defining the binding site. Tyr26 from both β2m molecules also help accommodate the ThT hydrophobic rings (Fig. 4c).

In order to house one or four ThT molecules, the β2m chains, which belong to two distinct DimC33 units, are differently juxtaposed. In particular, the two β2m molecules defining the 4 ThT site move about 20 Å apart compared to the 1 ThT site, to make room for the four ThT molecules. Thus, very limited direct interactions connect the two β2m molecules at the 4 ThT site, whereas more direct protein-protein contacts take place at the 1 ThT site. Despite such translational adjustments, the two ThT sites maintain very similar structural arrangements. The relative orientation of the two β2m chains defining the ThT sites is comparable, and the protein regions involved in ThT binding are conserved. More specifically, Tyr10, Tyr26 and Tyr63, the three central residues in the ABDE interface, cross diagonally the sheet and confer substantial surface hydrophobicity (Fig. 4). In particular, at both ThT sites Tyr10 establishes direct stacking interactions with ThT molecules, even though the ThT molecules do not share the same orientation in the two sites.

It must be noted that wide intermolecular packing contacts through the ABDE sheet are established also in the absence of ThT. In the structure of DimC33_low (present work) and in the hexameric H13F mutant^17^, highly hydrophobic association interfaces are built via facing ABDE sheets (surface areas of 805 and 970 Å^2^, respectively). Although such two ABDE assemblies are not superposable, the two interfaces are structurally similar and residues Tyr10, Tyr26 and Tyr63 are clustered in their hydrophobic cores (see Fig. 4d,e).

In summary, in the four ABDE packing interfaces examined (the 1 ThT and 4 ThT sites, and the ABDE interfaces in DimC33_low and in hexameric β2m) the orientations of each β2m monomeric chain may vary, the distances between protein chains are somewhat different, and the packing interactions between the ABDE sheets can be direct or mediated through ThT molecules. However, the overall conservation of protein-protein interaction surface, the binding of ThT, the size of the surfaces, the structural data and the marked hydrophobicity of the residues involved, all point at this sheet as a favored site for β2m association during oligomer formation.

## Discussion

Elucidating the structure and the underlying interactions of on-pathway oligomers that lead to amyloid aggregation is a crucial and challenging task. A structural characterization of the first steps of oligomer formation would be the key for a deeper understanding of the aggregation process, and potentially for the design of inhibitors hampering the oligomerisation process, and the related protein cytotoxicity.

We selected β2m as a model of amyloid aggregation, since the protein has been extensively characterized, and several structural determinants of β2m amyloidogenicity have been uncovered^7^. In order to shed light on the intermolecular interactions that drive the first steps of aggregation, a disulphide-linked β2m homodimer was designed according to a previously proposed strategy^19^. The engineered S-S bond was positioned in order to juxtapose two β2m monomers along the DD strand interface, which was observed as an intermolecular association region^17^,^19^. The mutation Ser33 to Cys is structurally conservative, and designed to bring together two β2m molecules through the DD strand interface, without altering its structure. From the *in vitro* biophysical data, and from the crystallographic analysis here reported, all evidences suggest that the mutation and the engineered disulphide bond do not affect β2m properties to a significant extent.

Under native conditions, we and others have shown that a minor population of wt β2m is oligomeric, mainly dimeric^14^,^23^. The observation that the disulphide bridge stabilizing DimC33 forms spontaneously in solution (in the absence of an added oxidizing agent) is indicative of the spontaneous juxtaposition of two facing C33 residues; it thus strongly suggests that the DD interface is the prevalent association interface underlying β2m dimerisation under such conditions.

**Figure 2:**
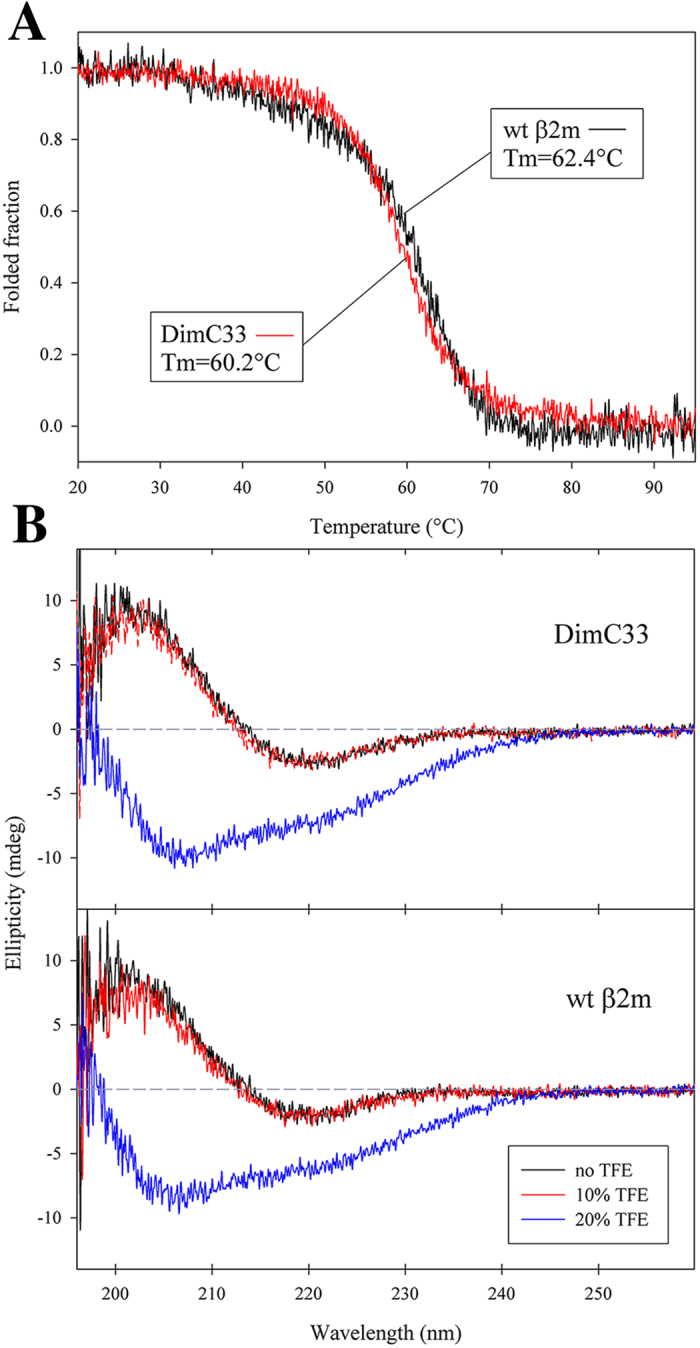
Thermal stability and Far-UV spectra analysis by circular dichroism. (**A**) Temperature ramps for DimC33 in red and wt β2m in black monitored at 202 nm, temperature slope 50 °C/h. Signals were reported as fractional variation of the total change. The melting temperature (Tm) values for DimC33 and wt β2m are shown in the graph. (**B**) Far-UV spectra for DimC33 and wt β2m in a buffer containing 100 mM sodium chloride, 50 mM sodium phosphate buffer pH 7.4, recorded under three different conditions: No TFE (black curves); in presence of 10% (v/v) TFE (red curves); and in 20% (v/v) TFE (blue curves).

**Figure 3:**
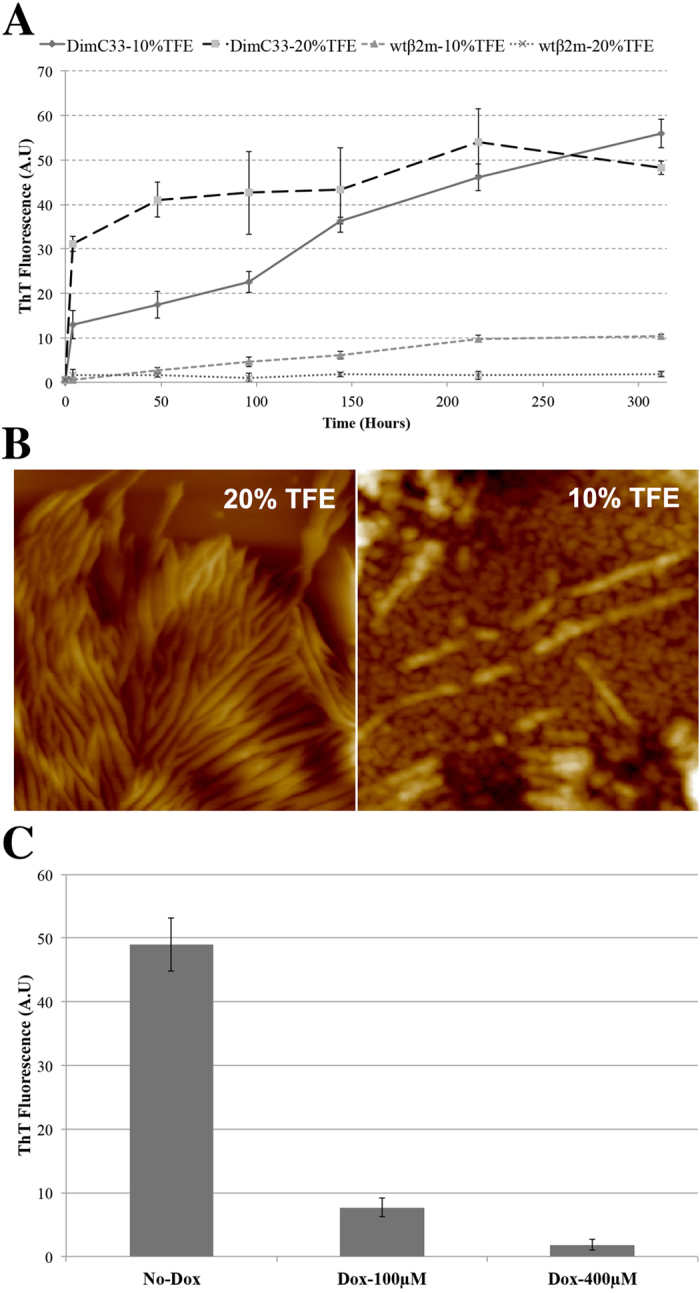
DimC33: fibrillogenesis at pH7.4. (**A**) Kinetics of DimC33 fibril formation in 100 mM NaCl, 50 mM Na phosphate buffer (pH 7.4) at 37 °C incubation for two weeks, monitored by measuring ThT fluorescence at 0, 4, 48, 96, 144, 216, 312 hours. Four samples were tested: DimC33_20% TFE and wtβ2m_20% TFE (1 mg mL^−1^ protein in 20% TFE); DimC33_10% TFE and wtβ2m_10% TFE (5 mg mL^−1^ protein in 10% TFE). Values represent the average of three independent experiments and error bars represent standard deviation (SD). (**B**) Tapping mode AFM images (height data) of DimC33 fibrillar aggregates obtained in 20% TFE (left) and 10% TFE (right). Scan size 500 nm, Z range: left, 37 nm; right, 15 nm. (**C**) DimC33 aggregation analysis monitored by ThT fluorescence after one week of incubation at 37 °C, using the same aggregation conditions as tested under DimC33_20% TFE in three conditions; 0 μM doxycycline, in the presence of 100 μM and 400 μM doxycycline. Values represent the average of three independent experiments and error bars represent SD.

**Figure 4:**
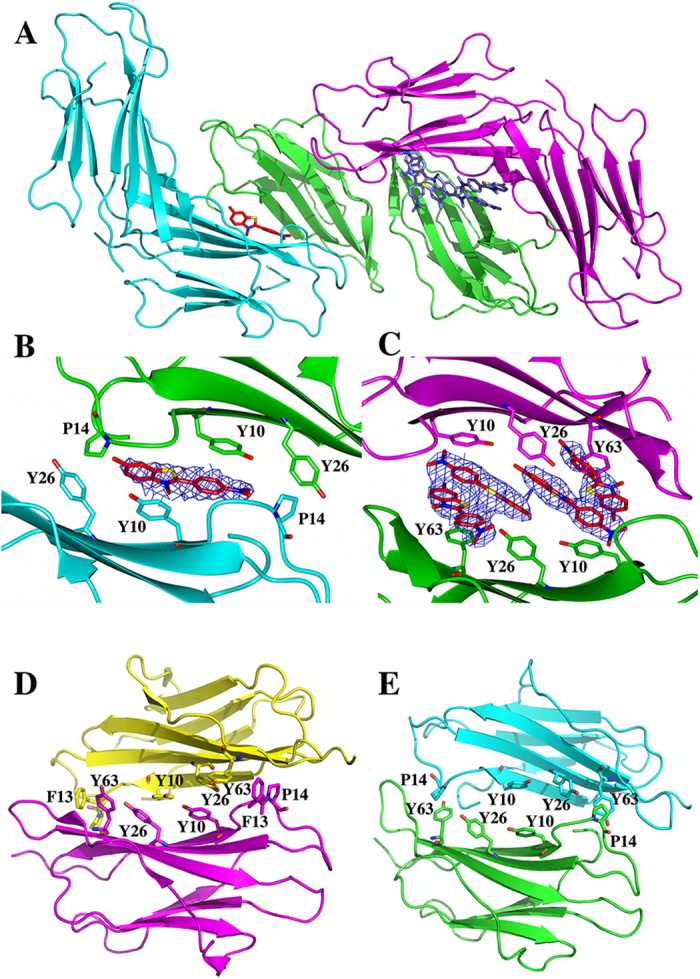
Crystal structures of DimC33_ThT and DimC33_low. (**A**) Cartoon representation of DimC33 assembly in the crystal structure of DimC33_ThT. The three DimC33 units are colored in cyan, green and magenta. Two ThT binding sites are visible: on the left side the 1 ThT site, on the right the 4 ThT site (the ThT molecules are colored in red). (**B**,**C**) A zoomed representation into the 1 ThT site (**B**) and into the 4 ThT site (**C**), showing ThT molecules sandwiched between the ABDE sheets of two adjacent DimC33 moieties, 2Fo-Fc omit electron density map at 1.5σ is clipped around ThT molecules. (**D**,**E**) Ribbon representation of the ABDE interface as observed (**D**) in the DimC33_low structure (pdb code: 4R9H); and (**E**) in the hexameric structure of H13F β2m (pdb code: 3CIQ).

The aggregation data show that DimC33 is much more amyloidogenic than wt β2m, even though its thermodynamic stability and its 3D structure are virtually identical to those of the wt protein. Using a standard β2m aggregation protocol in 20% TFE, we have shown that DimC33 aggregates abundantly, but, opposite to wt β2m, does not require fibril seeds for aggregation to start, an indication that DimC33 can spontaneously form the early oligomers required for the onset of fibrillogenesis. Analogously to wt β2m early oligomer formation^22^, doxycyclin proved to be an inhibitor of DimC33 aggregation. However, DimC33 was shown to aggregate also in 10% TFE, a condition under which β2m is properly folded (Fig. 2b). The data available to date indicate that the amyloidogenic intermediates display a native-like structure^13^,^14^,^24^; on these bases, a β2m variant, such as DimC33, which aggregates starting from non-denaturating conditions may prove to be an insightful system allowing the study *in vitro* of wt β2m early oligomerisation.

Recent reports indicate that the first wt β2m oligomer formed on the pathway to fibril association should be a head-to-head elongated dimer^5^,^13^,^14^,^15^. In particular, Rennella *et al.* also provided evidence that the dimer association interface should involve the BC loop region^14^, a finding that is in keeping with our previous data and with a recently published report^13^,^19^. Thus, the data here reported for DimC33, together with previous results, point to the DD strand interface as the firstly established protein-protein association interface during β2m aggregation. The increased aggregation propensity of DimC33 compared to wt β2m, would then be the result of the immediate availability of the first on-pathway amyloidogenic oligomer, that is DimC33.

Interestingly, the crystal structure of DimC33 in complex with ThT provides further information on the β2m aggregation path. ThT fluorescence is the most widely accepted spectroscopic method to discern cross-β fibrils from amorphous aggregate. The ThT molecules are held to intercalate the cross-β structure in the fibrils, resulting into a gain of fluorescence. Only two structures of ThT in complex with amyloidogenic proteins have been so far reported, and both concern β2m^25^. In one of these structures (pdb code: 3 MYZ) the authors suggest that ThT molecules are simply trapped between β2m molecules packed in the crystal lattice, while the second structure (pdb code: 3 MZT) presents technical issues that prevent a thorough discussion of ThT-β2m interactions (See Materials and Methods). In this context, the DimC33_ThT structure here reported adds substantially new information on ThT binding mode to an amyloidogenic protein.

In the DimC33_ThT complex structure, the ThT molecules are nestled between the ABDE sheets of two facing β2m molecules, in a highly hydrophobic environment. Notably, an extended ABDE interface between facing β2m molecules can also be observed in the absence of ThT in DimC33_low (this work), and in the hexameric β2m structure reported earlier^17^. The reciprocal orientation of the two facing ABDE β-sheets displays a high level of variability, but in all cases ABDE β-sheets face each other and the same three aromatic residues (Tyr10, Tyr26 and Tyr63) build the hydrophobic core of the association interface. Such variability can be accounted for by the presence of several Tyr residues and by the capability of hydrophobic interactions to form under different orientations of the contributing residues. The above observations point at the ABDE β-sheet as a second key interface involved in β2m oligomer formation, in keeping with a previous report supporting the role of surface aromatic residues in determining β2m amyloid propensity^26^. The structural adaptability of the ABDE surfaces may also explain the formation of several different dimeric/tetrameric building blocks, recently proposed by the EM reconstruction of mature β2m fibrils^16^.

In summary, our study presents an engineered β2m covalent dimer, DimC33, displaying the same biophysical properties of wt β2m in solution, such dimer mimics the first oligomer formed during β2m aggregation. Unlike wt β2m, DimC33 does not require the addition of seeds to start fibrillogenesis, and aggregates under conditions where β2m retains native secondary structure content. Given that *in vivo* wt β2m likely aggregates from a native-like folded species, DimC33 could then be considered the first model system to study β2m oligomerisation *in vitro*, resembling more closely the aggregation steps occurring *in vivo,* compared to the current available aggregation protocols based on denaturing conditions. Therefore DimC33 may also be an insightful system to test anti-oligomerisation inhibitors. Finally, the properties and the structures of DimC33 recapitulate previous evidences and indicate how oligomerisation may proceed during aggregation. The first dimeric oligomer would be built across the DD strand interface; at a later stage, the aromatic residues located on the ABDE sheet would contribute to the formation of a hydrophobic core for further association through a structurally adjustable ABDE interface.

## Materials and Methods

### Mutagenesis, expression and purification

The synthetic gene coding for the Ser33 to Cys β2m variant was purchased from Eurofins genomics. The gene of interest was inserted into pET21B expression vector and transformed into BL21 (DE3) *E. coli* strain. The mutated β_2_m was expressed and purified as previously reported^27^. At the end of the procedure, an additional purification step was introduced to separate the spontaneously formed DimC33 by size-exclusion chromatography (Superdex75 16/60 GE healthcare) and eluted with 20 mM Sodium Phosphate buffer pH 7.4.

### Circular dichroism

Circular dichroism (CD) studies were carried out on a Jasco J810 spectropolarimeter equipped with a Peltier system for temperature control and analyzed by means of Jasco software. All measurements were recorded at 0.1 mg/mL protein concentration in 100 mM sodium chloride, 50 mM sodium phosphate buffer pH 7.4 by using a 0.1 cm path length cuvette. Spectra were recorded in plain buffer or in buffer supplied with 10% or 20% TFE at 37 °C. Temperature ramp measurements were recorded at 202 nm from 20 to 95 °C (temperature slope 50 °C/h). Tm values were calculated from the first derivative of the recorded traces.

### Aggregation kinetics

DimC33 aggregation experiments were performed as unseeded reaction by incubating samples of 100 μL at 37 °C for two weeks, without agitation. The following aggregation conditions were tested: DimC33_20% TFE (1 mg mL^−1^ DimC33 in 20% TFE, 100 mM Sodium chloride, 50 mM Sodium phosphate buffer pH 7.4) according to a standard protocol^28^; DimC33_10% TFE (5 mg mL^−1^ DimC33 in 10% TFE, 100 mM Sodium chloride, 50 mM Sodium phosphate buffer pH 7.4). Unseeded aggregation experiments on wt β2m were also performed as controls. Aggregation kinetics were monitored by means of VARIAN Cary Eclipse spectrofluorimeter by measuring ThT fluorescence signal at excitation and emission wavelength of 445 and 480 nm, respectively^29^.

The inhibitory effect of doxycycline on DimC33 aggregation was studied under the same aggregation condition as employed for DimC33_20% TFE, by monitoring ThT fluorescence binding signal and inspecting sample morphology by AFM after one week of incubation at 37 °C without agitation. Two doxycycline concentrations were screened: DimC33_Doxy100 containing 100 μM Doxycycline; and DimC33_Doxy400 containing 400 μM Doxycycline. DimC33_20% TFE aggregation was used as control model. The measurements are the average of three independent experiments. Doxycycline, TFE and ThT were purchased from SIGMA-Aldrich.

### Atomic force microscopy

For AFM inspection, DimC33 samples were diluted 500-fold. A 10 μl aliquot was deposited on a freshly cleaved mica substrate and dried under mild vacuum. Tapping mode AFM images were acquired in air using a Dimension 3100 Scanning Probe Microscope equipped with a ‘G’ scanning head (maximum scan size 100 μm) and driven by a Nanoscope IIIa controller, and a Multimode Scanning Probe Microscope equipped with “E” scanning head (maximum scan size 10 μm), driven by a Nanoscope IV controller (Digital Instruments – Bruker). Single beam uncoated silicon cantilevers (type OMCL-AC160TS, Olympus) were used. The drive frequency varied between 270 and 330 kHz, the scan rate was between 0.5 and 0.8 Hz. Aggregate size was measured from the height in cross section of topographic AFM images.

### Crystallization and structure determination

DimC33 was crystallized under three different conditions by mixing equal amounts of 8 mg mL^−1^ protein and reservoir solutions containing: (i) DimC33_low: 25% v/v PEG 4 K, 0.2 M Imidazole-Malate pH7.0; (ii) DimC33_high: 28% PEG 400, 0.2 M Calcium chloride dihydrate, and 0.1 M Hepes sodium pH7.5; (iii) DimC33_ThT: 25% PEG 4 K, 0.1 M Sodium chloride, 5 mM ThT, and 0.1 M Hepes sodium pH8.0–8.2. All DimC33 crystals were grown at 20 °C, using the sitting drop vapor diffusion crystallization method. DimC33_low and DimC33_ThT crystals were cryo-protected in 20–33% glycerol solution containing the respective crystallization mother liquor, and cryo-cooled in liquid nitrogen. DimC33_low and DimC33_high X-ray diffraction data were collected at ID23-1 beam-line (ESRF Grenoble), and DimC33_ThT X-ray diffraction data were collected at P13 beamline (PETRA Hamburg). DimC33_low X-ray data were processed using MOSFLM^30^ and SCALA from the CCP4 software suite^31^ and XDS^32^ for DimC33_high and DimC33_ThT. The 3D structures of DimC33 were determined by molecular replacement using the Balbes software suite^33^. All structures were refined using Phenix.refine^34^ and REFMAC5^35^. A twin fraction of 0.46 was estimated by Xtriage^34^. Therefore an amplitude based twin refinement protocol was applied during DimC33_ThT refinement process in REFMAC5. Model building and structural analysis for all DimC33 structures were carried out with COOT^36^ and figures were prepared with Pymol and CCP4MG^37^,^38^. Omit-map has been generated by Phaser suite^34^ using the annealing option.

### Analysis of 3 MZT Structure

The structure with the PDB code 3 MZT was reported as a hexameric H13F β2m mutant in complex with Thioflavin (ThT)^25^. The structure was determined at 2.70 Å resolution. The three ThT molecules modeled in the complex are sandwiched at three intermolecular interfaces present in the hexameric assembly; each ThT molecule is presented in two alternative, but almost superimposable, conformations each at 0.5 occupancy. Temperature factors for ThT atoms range between 80 and 120 Å^2^ for each of the two conformations; neighboring protein residues, all modeled with full occupancy, display B-factors mainly in the 45–70 Å^2^ range.

When the 3 MZT structure was refined (in our lab, using data from the PDB database) in the absence of the ThT molecules, the residual ThT electron density for each binding site was reduced to a roughly spheroidal blob, much smaller than the size of a ThT molecule. Based on such observations, the 3 MZT molecular model of ThT interaction with β2m was not considered for the structural comparisons here reported.

#### Structure deposition

Structure factors and coordinates have been deposited in the Protein Data Bank under accession codes: 4R9H for DimC33_low, 4RAH for DimC33_high and 4RA3 for DimC33_ThT.

## Additional Information

**How to cite this article**: Halabelian, L. *et al.* A covalent homodimer probing early oligomers along amyloid aggregation. *Sci. Rep.* **5**, 14651; doi: 10.1038/srep14651 (2015).

## Figures and Tables

**Table 1:** Data collection and refinement statistics for DimC33 Structures.

	Structure (PDB entry)		
	DimC33-Low (4R9H)	DimC33-High (4RAH)	DimC33-ThT (4RA3)
Data collection			
Beam line	ID23-1 (ESRF)	ID23-1 (ESRF)	P13 (PETRA III, MX1)
Space Group	P4_1_22	C222_1_	P3_2_21
Unit cell constants (Å)	a = 68.84 b = 68.84 c = 200.04	a = 32.35 b = 47.70 c = 119.65	a = 80.0 b = 80.0 c = 177.7
Resolution (Å)	65.09–1.90 (2.00–1.90)	29.91–1.40 (1.42–1.40)	59.23–2.80 (2.95–2.80)
R_merge_^a^(%)	10.9 (94.0)	6.5 (49.5)	5.9 (113.9)
I/sig(I)	12.4 (2.2)	10.9 (1.9)	21.4 (2.0)
Completeness (%)	99.9 (99.6)	98.7 (86.6)	100.0 (100.0)
Redundancy	8.9 (8.3)	4.5 (3.4)	9.6 (10.0)
Unique reflections	38985 (5533)	18491 (787)	16940 (2432)
Refinement			
R_work_^b^(%)	21.17	16.62	17.07
R_free_^b^(%)	24.73	20.94	20.64
Rms Bond Length	0.013	0.019	0.009
Rms Bond Angle	1.402	1.919	1.453
Number of atoms	3480	1021	3407
Average B, all atoms (Å^2^)	42.0	17.0	114.0
Average B, Thioflavin (Å^2^)	–	–	102.8
Ramachandran plot			
Most favored region	375 (98.68%)	86 (100%)	367 (94.83%)
Allowed region	4 (1.05%)	0 (0%)	20 (5.17%)
Outliers	1 (0.26%)	0 (0%)	0 (0%)

Values in parenthesis are for the highest resolution shell.

^a^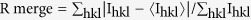 where I is the observed intensity and 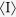 is the average intensity.

^b^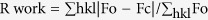 for all data except 5% which were used for Rfree calculation.

**Table 2:** The SSM superimposed RMS deviations for DimC33 structures with reference models DimC20, DimC50, hexameric H13F β2m and wt β2m (Å/Cα pairs superposed).

	DimC20 (3TLR)	DimC50 (3TM6)	H13F β2m (3CIQ)	wt β2m (1JF1)
DimC33-High (Mono)	0.62/99 Cα	0.59/98 Cα	1.05/97 Cα	0.97/98 Cα
DimC33-High (Dimer)	2.41/182 Cα	2.10/192 Cα	1.11/192 Cα	–
